# Silica/Polyethylene Glycol Hybrid Materials Prepared by a Sol-Gel Method and Containing Chlorogenic Acid

**DOI:** 10.3390/molecules23102447

**Published:** 2018-09-25

**Authors:** Michelina Catauro, Elisabetta Tranquillo, Alessia Salzillo, Lucia Capasso, Michela Illiano, Luigi Sapio, Silvio Naviglio

**Affiliations:** 1 Department of Engineering, University of Campania “Luigi Vanvitelli”, Via Roma 29, I-81031 Aversa, Italy; elisabetta.tranquillo@unicampania.it; 2 Department of Precision Medicine, University of Campania “Luigi Vanvitelli” Via L. De Crecchio 7, 80138 Naples, Italy; alessia_salzillo@libero.it (A.S.); luciacap93@hotmail.it (L.C.); michela.illiano@unicampania.it (M.I.); luigi.sapio@unicampania.it (L.S.); silvio.naviglio@unicampania.it (S.N.)

**Keywords:** sol-gel method, natural compounds, cell proliferation

## Abstract

Chlorogenic acid (CGA) is a very common dietary polyphenolic compound. CGA is becoming very attractive due to its potential use as preventive and therapeutic agent in many diseases, including cancer. Inorganic/organic hybrid materials are gaining considerable attention in the biomedical field. The sol-gel process provides a useful way to obtain functional organic/inorganic hybrids. The aim of this study was to synthesize silica/polyethylene glycol (PEG) hybrids with different percentages of CGA by sol-gel technique and to investigate their impact on the cancer cell proliferation. Synthesized materials have been chemically characterized through the FTIR spectroscopy and their bioactivity evaluated looking by SEM at their ability to produce a hydroxyapatite layer on their surface upon incubation with simulated body fluid (SBF). Finally, their effects on cell proliferation were studied in cell lines by direct cell number counting, MTT, flow cytometry-based cell-cycle and cell death assays, and immunoblotting experiments. Notably, we found that SiO_2_/PEG/CGA hybrids exhibit clear antiproliferative effects in different tumor, including breast cancer and osteosarcoma, cell lines in a CGA dependent manner, but not in normal cells. Overall, our results increase the evidence of CGA as a possible anticancer agent and illustrate the potential for clinical applications of sol-gel synthesized SiO_2_/PEG/CGA materials.

## 1. Introduction

**Figure 1:**
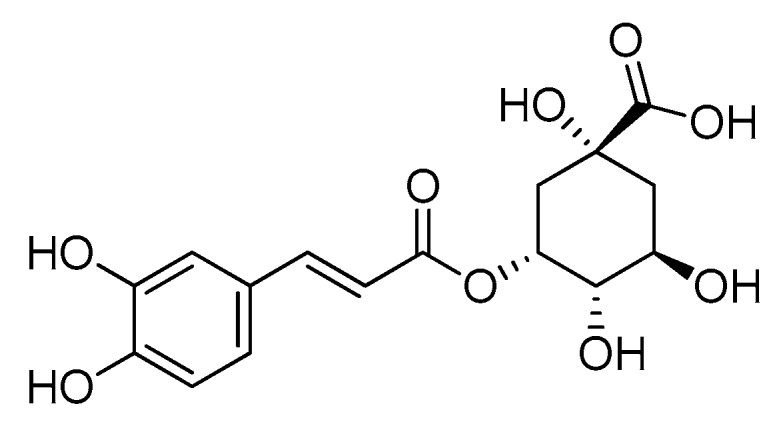
Molecular formula of chlorogenic acid (CGA).

Polyphenols are a heterogeneous group of natural substances, produced by the secondary metabolism of plants, that in relation to the chemical diversity that characterizes them, play different roles and display several pharmacological and therapeutic activities, which have been extensively studied in the area of oncology [1,2,3]. They are known for their positive action on human health, including targeted anticancer properties compared to conventional anticancer agents, as they have been described to show cytotoxicity mainly against neoplastic cells, but not in normal cells [4]. Chlorogenic acid (CGA, Figure 1) is a common polyphenolic compound present in different types of plants and fruits such as honeysuckle, Cortex Eucommiae, Semen Coffea Arabica, and green tea [5,6]. CGA is an ester formed from caffeic acid and quinic acid, containing both aromatic and aliphatic groups. It is becoming a very interesting and popular molecule because of its biological and pharmaceutical properties that confer it many human health benefits. It is widely known that CGA can act as an antibacterial, anti-inflammatory, and antioxidant agent, that also displays hypoglycemic and hypolipidemic activities [7,8,9,10,11,12]. Moreover, there is increasing evidence of a beneficial action of CGA in cancer prevention and treatment [13,14]. Notably, chlorogenic acid, as a polyphenol present in many dietary foods, is widely bioavailable in humans. However, its activities, as well as those of other polyphenolic compounds, are known to be limited to a short residence in the body [15]. In order to preserve and possibly enhance its bioactivity, in this study we aimed to produce silica/polyethylene glycol hybrids with different percentages of chlorogenic acid.

Many works about chlorogenic acid are reported in the literature [7,8,9,10], but only a few articles regarding its incorporation in supporting materials; for these reason SiO_2_/PEG/CGA hybrids can be considered as novel materials.

In the last few years, research on silica materials has received increasing attention [16]. In the literature it is reported that silica-based materials show high biological properties, furthermore, they provide successful solutions for soft and hard tissue regeneration [16]. It is known that the high biocompatibility of these materials and their positive biological effects are due to their reaction products able to promote cell-material interactions and cell invasion [16]. 

The addition of PEG improves the biocompatibility of the silica matrix by increasing the hydrophilicity of the materials and, therefore, improving cell adhesion and growth [17]. PEG is a versatile, biocompatible polymer that is used for many biomedical purposes [15,18,19], including polymer-based materials for drug delivery [20]. The reduction of toxicity and the extension of the circulation time of many drug nanocarriers, were improved by addition of PEG in the material [15,18,19]. In fact, SiO_2_/PEG hybrid materials have even been proposed as osteochondral regeneration matrices and in drug delivery applications [21].

Organic and inorganic hybrids are materials composed of intimately distributed organic and inorganic phases. The key aspect to synthesize hybrid materials is to maintain or improve the best properties of each of the components, eliminating or reducing their specific limitations. Hybrids can be synthesized by different methods, like solution blending or melt blending [22], but the sol-gel method is widely used in the formation and application of hybrid materials [23].

Using sol-gel technology it is possible to produce organic–inorganic hybrid materials, having as precursors monomers of silicon alkoxides or other metals. The sol-gel process allows one to prepare, at relatively low temperatures, materials based on inorganic oxides with the desired characteristics of hardness, chemical resistance, porosity and thermal resistance. The sol-gel method involves the hydrolysis of an alkoxide, the formation of a colloidal suspension and the evolution of the latter towards a gel in a condensation process [24]. The ability to form hydroxyapatite layers, thanks to the presence of numerous hydroxyl groups, allows one to obtain materials with a higher activity than those produced with other techniques [25].

The material properties of hybrids depend strongly on the nature and strength of the interactions between the organic and inorganic components. The classification of the hybrid materials have been proposed by Judenstein and Sanchez [26] according to the nature of their interactions: Class I consists of organic and inorganic compounds bonded through hydrogen, van der Waals, or ionic bonds, whereas in Class II, the organic and inorganic phases are linked through strong chemical bonds (covalent or polar covalent bonds).

Furthermore, these hybrids can also be classified as ‘green hybrids’ due to the addition of the CGA that is a natural compound. In fact, Unterlass [27] reported that hybrid materials that include at least one green step in their preparation or having at least one component of green origin are considered to be ‘green hybrids’.

In the current study, we have used the sol-gel method to build PEG-silica hybrid materials loaded with different contents (5 wt% and 10 wt%) of CGA, to test their capability to affect cancer cell proliferation compared to pure CGA.

## 2. Results and Discussion

### 2.1. Characterization of the Synthesized Materials

**Figure 2:**
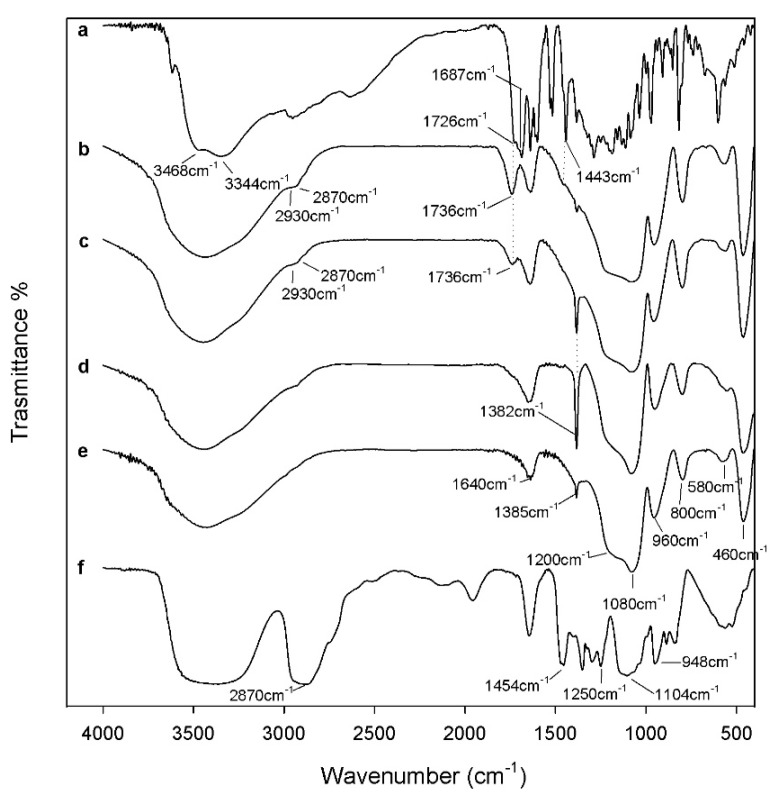
Representative FT-IR spectra a. CGA; b. SiO_2_/PEG_6wt%_/CGA_10wt%_; c. SiO_2_/PEG_6wt%_/CGA_5wt%_; d. SiO_2_/PEG_6wt%_; e. SiO_2_; f. PEG.

FT-IR analysis was used to identify the interaction among the different components embedded in the materials. The spectra of SiO_2_ with 6 wt% of PEG and different percentages of CGA are reported in Figure 2. 

**Figure 3:**
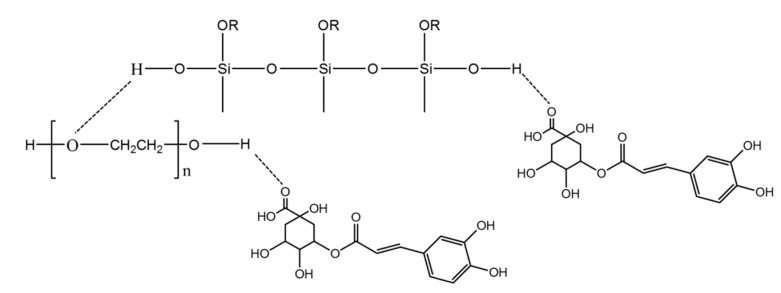
A graphical representation of the hybrids.

The typical peaks of silica in hybrid materials are more visible than those of the polymer, which is due to the low amount of PEG in the silica matrix. Comparing the spectra of hybrids (curves b, c) with pure silica (curve e), it is possible to observe, in all materials, peaks at 1080 cm^−1^, 1200 cm^−1^ and at 800 cm^−1^ that are ascribable to the typical asymmetric and symmetric Si–O stretching of silica matrix. The peaks at 460 cm^−1^ and the band at 950 cm^−1^ are assigned to bending Si–O–Si and Si–OH bond vibrations, respectively. The residual 4-membered siloxane rings in the silica network [28,29,30] are attributed to the band at 580 cm^−1^, while, the peak at 1385 cm^−1^ is due to N–O stretching [31] of residual nitrate anions from HNO_3_ used as catalyst in the synthesis procedure. The presence of hydrogen bonded –OH groups attached to the Si atoms [32] is confirmed by the position and the shape of the peaks at 3450 cm^−1^ and at 1640 cm^−1^. The low addition of polymer (6 wt%) in silica matrix allows to observe, in the hybrid spectra (curve b–d), only the bands at 2930–2870 cm^−1^ due to PEG methylene C–H stretching and bending, respectively. The different shape and position of the broad band about at 3400 cm^−1^ and of the Si–OH band suggest the formation of H-bonds between the –OH groups of the inorganic phases and ethereal oxygen atoms (H-bond donors) or terminal –OH in the PEG chains [33]. The hybrids’ spectra were compared with the pure CGA spectra (curve a). When the CGA is added at lower concentration in the hybrids, it is not possible to observe all characterized peaks between 1400 and 1500 cm^−1^. In particular, only in the spectra of the SiO_2_/PEG_6wt%_/CGA_10wt%_ is visible a little intense band at 1443 cm^−1^ which corresponds to phenyl ring stretch. Furthermore, in hybrids spectra is observed the displacement of the stretching C=O vibration band from 1726 cm^−1^ to 1736 cm^−1^ because the establishment of H-bonds with the SiO_2_ inorganic matrix occurs [34]. Furthermore, the intensity of this peak (1736 cm^−1^) increases in function of CGA amount. The broadening of the SiO_2_ strong band at 1080 cm^−1^ and of the shoulder at 1200 cm^−1^ suggest the presence of drug in inorganic matrix, because in this spectral region, the intense signals of the phenyl ring and C–O–C bonds in pure CGA (see curve a) are present [34]. The OH bending of the phenol function peak at 1385 cm^−1^ [34] are visible in both hybrids spectra with CGA, but a different intensity is observed. Probably, in SiO_2_/PEG_6wt%_/CGA_5wt%_ this peak is more intense than others, because the drug at low concentration is more embedded and linked within it by the formation of -H bonds. Whereas, when the higher amounts of CGA are added in silica/PEG (curve b) a part of the CGA cannot forms H-bonds because all hydroxyl groups of silica are already involved in H-bonds [35].Therefore, the FTIR results confirm that all components are linked to each other by hydrogen bonds (Figure 3) and the presence of polymer and CGA does not influence their interaction with SiO_2_ (curve e).

### 2.2. Bioactivity Test

The materials have been soaked in simulated body fluid (SBF) for 7, 14 and 21 days, and after this exposure time, the nucleation of the hydroxyapatite on the surfaces of all samples was detected by SEM images. No difference was observed in the distribution and amount of hydroxyapatite. In fact, the surfaces of all samples appear covered by a precipitate with the globular shape typical of hydroxyapatite [36].

**Figure 4:**
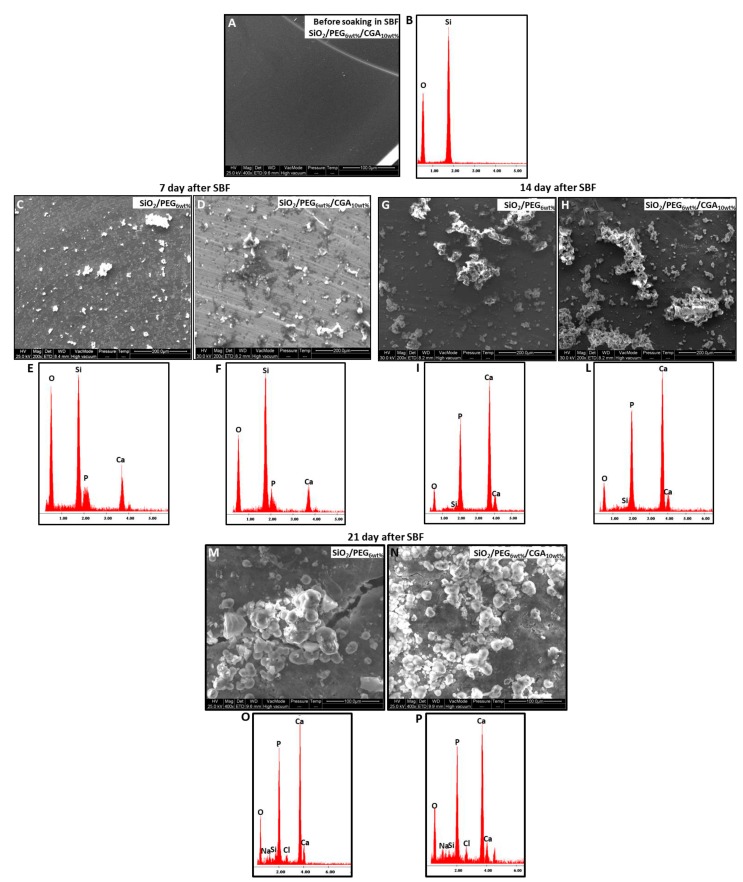
SEM micrographs of (**A**) a representative SiO_2_/PEG_6wt%_/CGA_10wt%_ hybrid (**B**) EDX analysis of SiO_2_/PEG6wt%/CGA10wt% hybrid before exposure to SBF, (**C**) SiO_2_/PEG_6wt%_ and (**D**) a representative SiO_2_/PEG_6wt%_/CGA_10wt%_ hybrid after 7 days in SBF; (**E**) EDX analysis of SiO_2_/PEG_6wt%_ hybrid and (**F**) EDX analysis of SiO_2_/PEG_6wt%_/CGA_10wt%_ hybrid after 7 days in SBF. (**G**) SiO_2_/PEG_6wt%_ and (**H**) a representative SiO_2_/PEG_6wt%_/CGA_10wt%_ hybrid after 14 days in SBF; (**I**) EDX analysis of SiO_2_/PEG_6wt%_ hybrid and (**L**) EDX analysis of SiO_2_/PEG_6wt%_/CGA_10wt%_ hybrid after 14 days in SBF. (**M**) SiO_2_/PEG_6wt%_ and (**N**) a representative SiO_2_/PEG_6wt%_/CGA_10wt%_ hybrid after 21 days in SBF; (**O**) EDX analysis of SiO_2_/PEG_6wt%_ hybrid and (**P**) EDX analysis of SiO_2_/PEG_6wt%_/CGA_10wt%_ hybrid after 21 days in SBF.

SEM micrographs of the materials before soaking in SBF (Figure 4A) show that are homogeneous and no phase separation was visible even at high magnifications, instead when the material was soaked in SBF, it was possible to observe that the surfaces of materials appeared covered by a precipitate with the globular shape typical of hydroxyapatite, also in absence of CGA.

Therefore, the SEM micrographs of the SiO_2_/PEG_6wt%_ and SiO_2_/PEG_6wt%_/CGA_10wt%_ hybrids are compared (Figure 4C–N) to prove that the presence of the drug does not affect the bioactivity of the materials. 

The SEM micrographs (Figure 4C,D,G,H) show a less intense hydroxyapatite layer on the surface of the different materials after 7 and 14 days compared with the hybrids exposed for 21 days to SBF(Figure 4M,N).

The energy-dispersive X-ray (EDX) microanalysis of the hybrids after 7, 14 and 21 days in SBF was compared with that of the same samples without exposure to SBF (Figure 4E,F,I,L,O,P). In Figure 4B it is possible to observe only the peaks of the elements that formed the hybrids, while, the other EDX confirmed that the globules consist of Ca and P that have a different atomic ratio depending on the days of exposure. After 21 days in SBF the atomic ratio is equal to 1.67, furthermore, the others peaks are residuals from sample preparation and SBF solution.

The presence of Si–OH groups on their surface induce the hydroxyapatite nucleation when soaked in SBF it is due to the interaction of these groups with the Ca^2+^ ions present in the fluid leading to an increase of positive surface charge. The Ca^2+^ ions combine with the negative charge of the phosphate ions to form amorphous phosphate, which spontaneously transforms into hydroxyapatite [Ca_10_(PO_4_)_6_(OH)_2_] [37].

**Figure 5:**
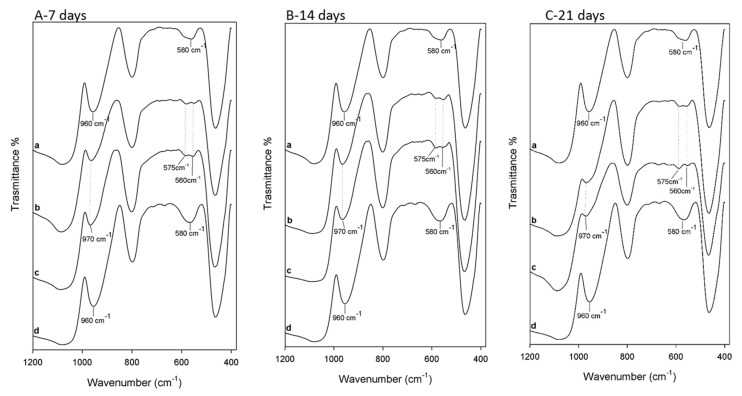
FT-IR spectra of (**A**) a. SiO_2_/PEG_6wt%_/CGA_5wt%_ not soaked in SBF; b. SiO_2_/PEG_6wt%_/CGA_5wt%_ c. SiO_2_/PEG_6wt%_/CGA_10wt%_ after 7 days of exposure to SBF d. SiO_2_/PEG_6wt%_/CGA_10wt%_ not soaked in SBF. (**B**) a. SiO_2_/PEG_6wt%_/CGA_5wt%_ not soaked in SBF; b. SiO_2_/PEG_6wt%_/CGA_5wt%_ c. SiO_2_/PEG_6wt%_/CGA_10wt%_ after 14 days of exposure to SBF d. SiO_2_/PEG_6wt%_/CGA_10wt%_ not soaked in SBF. (**C**) a. SiO_2_/PEG_6wt%_/CGA_5wt%_ not soaked in SBF; b. SiO_2_/PEG_6wt%_/CGA_5wt%_ c. SiO_2_/PEG_6wt%_/CGA_10wt%_ after 21 days of exposure to SBF d. SiO_2_/PEG_6wt%_/CGA_10wt%_ not soaked in SBF.

To confirm the presence of hydroxyapatite layer on the samples surface, FT-IR analysis was used. The Figure 5 shows the spectra of the hybrids after 7, 14 and 21 days of exposure to SBF (curve b, c) compared to the spectra of the same materials not soaked in SBF (curve a, d). The spectra after 7 (Figure 5A, 14 (Figure 5B) and 21 days (Figure 5C) shows the same results. The interaction of the hydroxyapatite layer with the –OH groups of the silica matrix was suggested by the displacement of Si–OH band, from 960 cm^−1^ to 970 cm^−1^. Furthermore, the split of the band at 580 cm^−1^ in two new ones at 575 cm^−1^ and 560 cm^−1^, was observed. These bands are due to the stretching of the –OH groups of hydroxyapatite and the vibrations of the ${\mathrm{PO}}_{4}^{3-}$ groups caused by the formation of the hydroxyapatite precipitate [38,39]. The intensity of the typical peaks of hydroxyapatite in the spectra of the hybrids after 7 and 14 days (Figure 5A,B) is lower compared with those at 21 days, these results confirms the SEM data.

**Figure 6:**
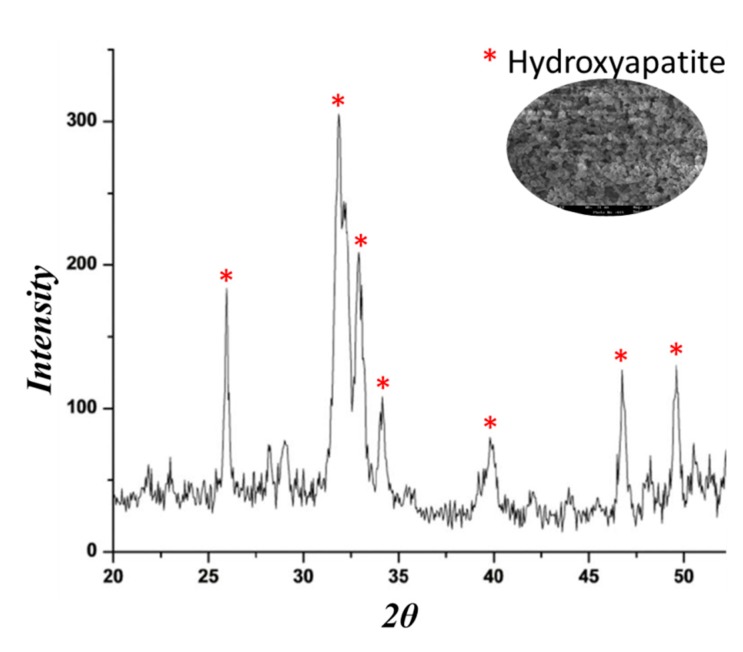
A representative XRD of SiO_2_/PEG_6wt%_/CGA_10wt%_ soaked in SBF solution for 21 days.

The hydroxyapatite nucleation on the hybrids surface was investigated, also, by XRD measurement. Figure 6 shows the intense peaks of crystalline hydroxyapatite confirming its formation after soaking in SBF for 21 days. Those results suggest that the materials surface is covered by a hydroxyapatite layer and it is very thick.

### 2.3. In Vitro Anticancer Activity of CGA-Containing SiO_2_/PEG Hybrid Materials

**Figure 7:**
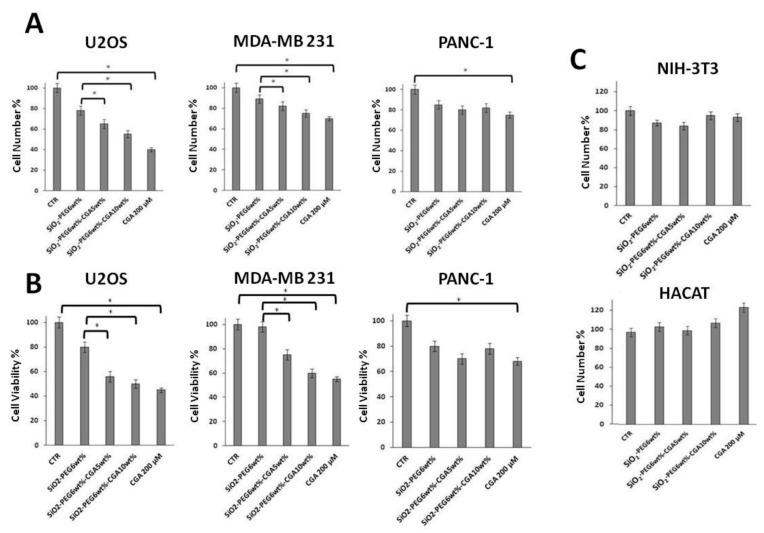
Effects of SiO_2_/PEG hybrid 0.5 mg/mL, SiO_2_/PEG_6wt%_/CGA_5wt%_ hybrid 0.5 mg/mL, SiO_2_/PEG_6wt%_/CGA_10wt%_ hybrid 0.5 mg/mL, pure CGA 200 µM, and doxorubicin 0.1 µM on the distribution of U2OS cells in cell cycle phases and on cell death. U2OS cells were cultured in presence or not of doxorubicin for 24 h and of all the other indicated compounds for 48 h. Subsequently, cell cycle phases (Panel **A**), sub-G1 (Panel **B**) or uptaking PI+ dead cells (Panel **C**) were evaluated by Flow Cytometry. Quantitative data from three independent experiments indicating the percentage of sub-G1, G1, S, and G2/M cells or the percentage of dead cells are reported. The means and S.D. are shown. * *p* < 0.05 vs. the control untreated cells (Panel **A**) or vs. the indicated samples (Panels **B** and **C**).

The PANC-1, MDA-MB-231 and U2OS cell lines are well-established and widely used model systems of human pancreatic, triple negative breast cancer, and osteosarcoma cancer cells, respectively [40,41,42,43,44,45]. In order to evaluate the possible antitumor properties of CGA-containing SiO_2_/PEG hybrids, we firstly looked at their impact on the proliferation of PANC-1, MDA-MB-231 and U2OS cells. We used the CGA-free SiO_2_/PEG hybrid as a reference standard. In our experiments we also included a commercial source of pure CGA as internal positive control. To that purpose, cells were grown for 48 h in the absence (control cells) or presence of the following compounds: CGA-free SiO_2_/PEG hybrid, 5% CGA-containing SiO_2_/PEG hybrid, 10% CGA-containing SiO_2_/PEG hybrid, pure CGA. After treatments, direct cell number counting and conventional tetrazolium-based (MTT) assays were performed (Figure 7).

In agreement with previous results [40,41], in Figure 7A,B it is shown that proliferation of MDA-MB-231 cells was inhibited by CGA. In addition, according to its suggested anticancer action, pure CGA treatment resulted in the reduction of the number of PANC-1 and U2OS cells, too. Notably, the antiproliferative effect in osteosarcoma U2OS cells was impressive with an inhibition of about 60%, whereas in PANC-1 and MDA-MB-231 cells it was of about 20% and 30%, respectively, suggesting a different sensitivity of the three cell lines to CGA, being the U2OS cells much more susceptible. Interestingly, Figure 7A,B shows that the CGA-containing hybrid materials do have antiproliferative effects, too. More in details, both 5% and 10% CGA-containing hybrids clearly reduce the number of MDA-MB-231, and, even more, U2OS cells, whereas only minimal effects could be seen on PANC-1 cells. Moreover, in agreement with previous findings [46,47], a small (≈15%) decrease of the cell number in response to CGA-free SiO_2_/PEG hybrids is evident as compared to control cells (grown in the absence of materials), according to a growth impairment/minimal toxicity that can be induced by such materials [48].

To increase the specificity and reliability of our data, we looked also at the impact of our compounds on the proliferation of NIH-3T3 fibroblasts and HACAT keratinocytes [48,49]. Very interestingly, Figure 7C clearly shows that CGA-containing SiO_2_/PEG hybrids, as well as pure CGA, do not have antiproliferative effects in such normal, not cancer, cells. Altogether, the above data confirm and extent the evidence that CGA can act as anticancer agent with discrete antiproliferative effects, depending on cell type, and indicate that sol-gel synthesized SiO_2_/PEG/CGA materials might be functional tools with anticancer activity, not toxic against normal cells.

### 2.4. Antiproliferative Effects by CGA-Containing SiO_2_/PEG Hybrid Materials are Depending Mainly on Cell Death Induction

**Figure 8:**
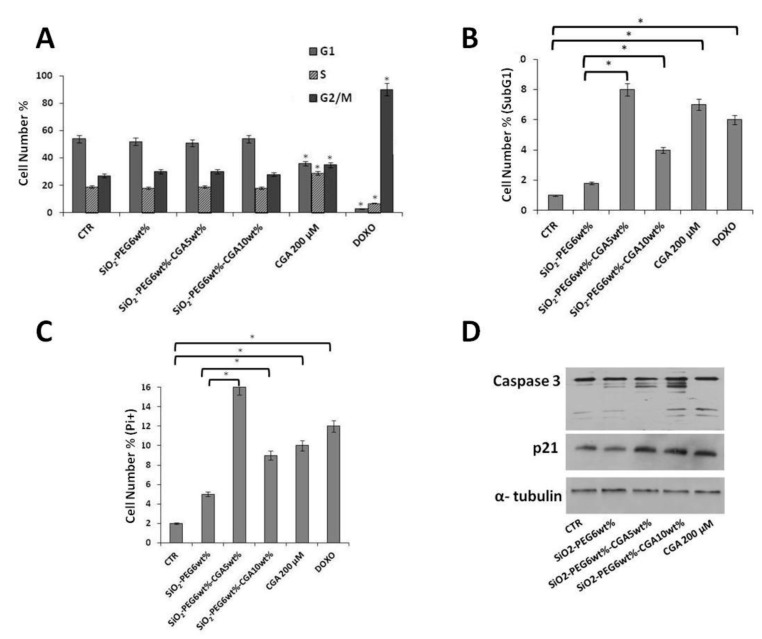
Effects of CGA-free SiO_2_/PEG hybrid 0.5 mg/mL, 5%CGA-containing SiO_2_/PEG hybrid 0.5 mg/mL, 10%CGA-containing SiO_2_/PEG hybrid 0.5 mg/mL, pure CGA 200 µM, and doxorubicin 0.1 µM on the distribution of U2OS cells in cell cycle phases and on cell death. U2OS cells were cultured in presence or not of doxorubicin for 24 h and of all the other indicated compounds for 48 h. Subsequently, cell cycle phases (Panel **A**), sub-G1 (Panel **B**) or uptaking PI+ dead cells (Panel **C**) were evaluated by Flow Cytometry. Quantitative data from three independent experiments indicating the percentage of sub-G1, G1, S, and G2/M cells or the percentage of dead cells are reported. The means and S.D. are shown. * *p* < 0.05 vs. the control untreated cells (Panel **A**) or vs. the indicated samples (Panels **B** and **C**). In (Panel **D**), 30 μg of cell extracts were subjected to SDS-PAGE and blotted with antibodies against the indicated proteins. The image is representative of immunoblotting analysis from two different cellular preparations with similar results.

To further explore the growth inhibitory effect of CGA-containing SiO_2_/PEG hybrids on cancer cells, the distribution of U2OS cells in the cell cycle phases was evaluated by flow cytometric analysis of propidium iodide-stained cells (Figure 8A). We also looked at the proportion of cells with hypodiploid DNA content (sub-G1 population), characteristic of cells having undergone DNA fragmentation, which is a biochemical hallmark of cell death (Figure 8B). First of all, Figure 8A shows that treatment with 0.1 µM doxorubicin for 24 h (which we used as internal positive control of “cell cycle” modifier, in such settings) causes a strong G2/M accumulation, as expected [50]. Moreover, Figure 8A also shows that the distribution of the pure CGA-treated cells is markedly different compared to control untreated cells, whereas only minimal changes in cell cycle phases are observed in response to CGA-containing SiO_2_/PEG hybrids. Interestingly, a sub-G1 population appeared in response to both pure CGA and CGA-containing SiO_2_/PEG materials, consistent with cell death (Figure 8B). This was further confirmed by measurement of cell death by more sensitive PI uptake assay [51]. In Figure 8C it is clearly shown that in presence of CGA and CGA-containing SiO_2_/PEG materials the percentage of dead cells population is clearly increased, compared to control untreated and CGA-free SiO_2_/PEG composite samples, respectively.

Overall, the above data suggest that CGA-containing SiO_2_/PEG materials, although their impact on cell cycle progression cannot be definitively ruled out, can have anticancer activity mainly by inducing cell death in osteosarcoma cells. Additionally, our data indicate that pure CGA, consistent with its anticancer role, can both inhibit the cell cycle and induce cell death in osteosarcoma cells.

To gain further insight into the effects of CGA-containing SiO_2_/PEG hybrid materials on the cell cycle and cell death, we started to examine the expression of some relevant proteins involved in cell cycle and cell death, by immunoblotting analysis. Initial data are encouraging and seem to suggest that consistent variations of protein levels, such as cyclin-dependent kinase inhibitor p21 and caspase 3, occur in response to our compounds (Figure 8D).

## 3. Materials and Methods

### 3.1. Sol-Gel Synthesis of the Materials

The sol-gel method was used to prepare SiO_2_/PEG/CGA hybrid materials. Tetraethyl orthosilicate (5.6 mL) (TEOS; Si(OC_2_H_5_)_4_; Sigma-Aldrich, St. Louis, MO, USA), used as metal alkoxide precursor, was added in a solution of HNO_3_ (1.0 mL) (≥65%, Sigma-Aldrich), distilled water (2.2 mL) and pure ethanol (8.8 mL) (99.8% Sigma-Aldrich) to obtain the silica matrix. The microstructural properties of the inorganic matrix are affected by acid pH, as well as the H_2_O/alkoxide molar ratio [52].

**Figure 9:**
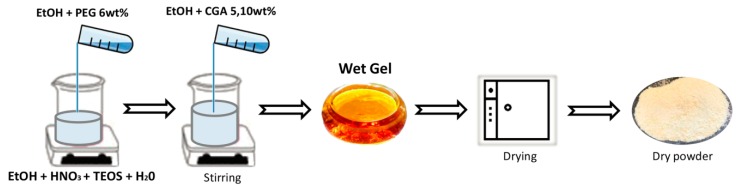
Flowchart of the sol-gel procedure used.

To the silica sol, a solution of PEG (MW = 400, Sigma-Aldrich) (C = 6 wt%) solubilized in 3.5 mL of ethanol (99.8%, Sigma-Aldrich) was added and the mixture stirred for 15 min. Subsequently, solutions of CGA (95%, Sigma-Aldrich) of different concentrations (C = 5, 10 wt%) dissolved in 3.5 mL of ethanol (99.8%, Sigma-Aldrich) was slowly added to the silica and PEG solution while stirring and kept stirring for 15 min. After 20 min the various gels were air-dried at 40 °C for 24 h to obtain a dry powders and to remove the residual solvent to prevent the thermal degradation of both polymer and drug. In the gels the molar ratios of the reagents are: EtOH/TEOS = 6.2 TEOS/HNO_3_ = 1.7, H_2_O/TEOS = 6. A flow chart of the sol-gel procedure used is shown in Figure 9.

### 3.2. Study of the Material Structure

Fourier transform infrared spectroscopy (FT-IR) was used to evaluate the chemical composition of the materials and the interaction between their components. To analyze the various materials, the sample powder was pressed into a cylindrical holder using a Specac manual hydraulic press. The obtained disks, with a weight of 200 mg, with a diameter of 13 mm, a thickness of 2 mm were prepared using 1 wt% of sample in KBr. Transmittance spectra were recorded in the 400–4000 cm^−1^ region using a Prestige 21 (Shimadzu, Kyoto, Japan) system, equipped with a Deuterated Tryglycine Sulphate with potassium bromide (DTGS KBr) window detector, with a resolution of 4 cm^−1^ (45 scans) and the Prestige software (IR Solutions, Shimadzu, Kyoto, Japan) was used to analyze the FT-IR spectra.

### 3.3. Bioactivity Test

The hybrid powders were obtained using an agate mortar. As reported by Kokubo [36], to evaluate their bioactivity, the materials were soaked for 7, 14 and 21 days in a simulated body fluid (SBF); the ion concentration in SBF is nearly equal to that in human blood plasma and the solution temperature fixed at 37 °C. The reaction of hydroxyapatite nucleation is affected by the total surface area of the material exposed to SBF and its volume; thus, a constant ratio was maintained, according to literature [36,53,54]. Then, the SBF solution containing the materials was exchanged every 2 days, to minimize the decrease of the ionic species due to the formation of biominerals in the SBF. After 7, 14 and 21 days the samples, were removed from the SBF and then air-dried in a desiccator. To evaluate the ability to form an apatite layer on their surfaces, Quanta 200 SEM (FEI, Eindhoven, The Netherlands), equipped with energy-dispersive X-ray (EDX) and Fourier transform infrared spectroscopy (FT-IR) were used. Furthermore, the apatite layer on the hybrids materials was analyzed by XRD analysis using a Philips 139 diffractometer equipped with a PW 1830 generator, tungsten lamp and Cu anode, where the source 140 of X-ray is given by a Cu-Kα radiation (λ = 0.15418 nm).

### 3.4. Cell Culture and Treatments

The human pancreatic cancer PANC-1 and the human osteosarcoma U2OS cell lines were purchased from the American Type Culture Collection (ATCC, Manassas, VA, USA), instead the human keratinocytes HaCat, the mouse fibroblast NIH-3T3, and MDA-MB-231 human triple negative breast cancer cell lines were obtained from the American Type Culture Collection (Rockville, MD, USA). The cells were cultured in Dulbecco’s Modified Eagle’s Medium (DMEM) supplemented with 10% fetal bovine serum (FBS), 2 mM glutamine, 100 U/mL penicillin, 100 mg/mL streptomycin and cultured at 37 °C in a 5% CO_2_ humidified atmosphere. Generally, cells were split (5 × 10^5^/10 cm plate, 6 × 10^4^/6-well plate) and grown in 10% serum containing medium for 24 h, then the medium was removed and incubated with 10% FBS fresh medium (Time 0), in the absence (control cells) or in the presence of the CGA-free SiO_2_/PEG_6wt%_ hybrid, SiO_2_/PEG_6wt%_ containing 5 wt% CGA hybrid, SiO_2_/PEG_6wt%_ containing 10 wt% CGA hybrid (all of them at 0.5 mg/mL concentration), a commercial source of pure CGA (95%, Sigma-Aldrich) at 200 µM concentration. Times and concentrations are indicated in the text and figures. After treatment, adherent and floating cells were collected by trypsinization and centrifugation respectively and counted.

### 3.5. Cell Viability Assay

Cell viability assay was performed as described previously [55]. In brief, cells were seeded in 96-well plate (3 × 10^3^ cells/well) and grown in 10% PBS-containing medium for 48 h at 37 °C. Viable cells were determined by the 3-[4-dimethylthiazol-2-yl]-2,5-diphenyltetrazolium bromide (MTT) assay and cell viability was evaluated by adding MTT solution in PBS to a final concentration of 0.5 mg/mL. Then, the plates were incubated for 4 h at 37 °C and the MTT-formazan crystals were solubilized in a solution of 4% 1N isopropanol/hydrochloric acid at room temperature on horizontal table shaking for 20 min. The absorbance was measured at 570 nm with Bio-Rad 550 microplate reader (Bio-Rad Laboratories, Milan, Italy). Cell viability was expressed as a percentage of absorbance values in treated samples compared to that of control (100%). MTT experiments were repeated three times (in replicates of 6 wells for each data point in each experiment). Data are presented as means ± standard deviation for a representative experiment.

### 3.6. Evaluation of Cell Cycle Phases by Flow Cytometry

The cells were treated as previously described in “Cell culture and treatments”, and fixed in 70% ice-cold ethanol/PBS and stored overnight at 4 °C. Successively, the cells were spun-down at 400× *g* for 5 min, washed two times with ice-cold PBS, centrifuged again and re-suspended in 1 mL PI staining solution (50 μg/mL PI and 100 μg RNase A in PBS). Then the samples were moved to 5-mL BD-Falcon tubes and incubated at 4 °C until assayed. Flow cytometry analysis was performed with a FACSCalibur flow cytometer (Becton Dickinson, Franklin Lakes, NJ, USA), at least 20 K events for each samples were acquired. Finally, the percentage of G1, S, G2/M phase and also for the sub-G1 events were defined by ModiFIT Cell Cycle Analysis software [50].

### 3.7. Cell Death Assay by Propidium Iodide Uptake and Flow Cytometry Analysis

Cell death was evaluated as previously described [41]. Due to mechanisms of death (apoptosis, necrosis, autophagy etc.), plasma membranes generally become permeable to PI. In fact, changing of plasma membrane permeability is the essential condition to allow to Propidium Iodide (PI) to bind DNA. Thus, PI uptake is usually considered as a method to discriminate dead cells (PI positive cells) from live cells. The cells were cultured in 6-well plates (10 × 10^4^ cells/well) and grown for 24 h. Successively, the medium was removed and cells were treated for times, concentrations and modalities reported in “Results” section. The cells were collected and incubated with PI-FACS buffer containing 0.5 mg/mL of PI in PBS and analyzed by flow cytometry.

### 3.8. Preparation of Cell Lysates

Cell extracts were prepared as previously described [55]. After treatment, the cells were collected as previously described in “Cell culture and treatments”; 3–5 volumes of RIPA buffer (PBS, 1% NP-40, 0.5% sodium deoxycholate, 0.1% SDS) containing 10 μg/mL leupeptin, aprotinin, and 1 mM phenylmethylsulfonyl fluoride (PMSF) were added to the cells. After the cells were incubated on ice for 1 h and centrifuged at 18,000× *g* in an Eppendorf microcentrifuge for 15 min at 4 °C; the supernatant (SDS total extract) was recovered. Some aliquots were diluted in 4× Leammli buffer, boiled and stored for immunoblotting analysis while other aliquots were used for protein quantification according to the Bradford method.

### 3.9. Western Blot Analysis

The proteins from whole extracts (typically 20–40 μg) were separated by sodium dodecyl sulphate-polyacrylamide gel electrophoresis (SDS-PAGE) and transferred onto nitrocellulose membrans (Schleicher & Schuell, Dassel, Germany) by a Mini Trans-Blot apparatus BioRad (Hercules, CA, USA). The membranes were washed in TBST (10 mM Tris, pH 8.0, 150 mM NaCl, 0.05% Tween 20), and blocked with TBST supplemented with 5% nonfat dry milk. After the membranes were incubated overnight with different primary antibodies in TBST and 5% nonfat dry milk. The day after, the membranes were incubated with horseradish peroxide-conjugated secondary goat anti-rabbit or anti-mouse antibodies, conjugated with horseradish peroxidase (BioRad). Enhanced chemiluminescence detection reagents were used as a detection system (ECL) according to the manufacturer’s instructions (Amersham Biosciences, Little Sharfont, UK).

### 3.10. Statistical Analysis

Most experiments were performed at least three times with replicate samples. Data are plotted as mean ± SD (standard deviation). Differences in mean between different experiments were calculated using analysis of variance (ANOVA) plus Bonferroni’s *t*-test. *p* values of less than 0.05 were considered significant.

## 4. Conclusions

The possibility to improve the biological characteristics of relevant natural compounds by using the versatility of the sol-gel method is considered an intriguing challenge. Chlorogenic acid (CGA), is an increasingly attractive natural phenolic compound, due to its proposed health protective properties, including anticancer activity. Here, we demonstrate that CGA, at concentrations not toxic to normal cells, has a strong in vitro antitumor activity against osteosarcoma cells. In addition, we provide clear evidence that silica/polyethylene glycol (PEG) hybrid materials embedded with different percentages of CGA synthesized by sol-gel technology, have antiproliferative activities on cancer cells, mostly evident in osteosarcoma cells, with discrete effects depending on cell type, and mainly due to cell death induction. Interestingly, our data are in full accord with recent findings showing that CaP composites containing CGA, in the presence of alginate as a polymer template, (i.e., other organic-inorganic hybrids containing CGA) have anticancer activity on osteosarcoma cells [15]. Regarding the underlying molecular mechanisms by which CGA and CGA-containing silica/polyethylene glycol hybrids induce inhibition of cancer cell proliferation, they need to be understood and will be investigated further in future studies. Altogether, our findings enrich the evidence of CGA as a potential anticancer agent and outline the rationale for possible clinical applications of sol-gel synthesized SiO_2_/PEG/CGA hybrid materials.
